# Recent Advances on Phagocytic B Cells in Teleost Fish

**DOI:** 10.3389/fimmu.2020.00824

**Published:** 2020-05-27

**Authors:** Liting Wu, Zhendong Qin, Haipeng Liu, Li Lin, Jianmin Ye, Jun Li

**Affiliations:** ^1^Guangdong Provincial Key Laboratory for Healthy and Safe Aquaculture, Institute of Modern Aquaculture Science and Engineering, School of Life Sciences, South China Normal University, Guangzhou, China; ^2^Guangzhou Key Laboratory of Aquatic Animal Diseases and Waterfowl Breeding, Guangdong Provincial Water Environment and Aquatic Products Security Engineering Technology Research Center, College of Animal Sciences and Technology, Zhongkai University of Agriculture and Engineering, Guangzhou, China; ^3^State Key Laboratory of Marine Environmental Science, State-Province Joint Engineering Laboratory of Marine Bioproducts and Technology, Xiamen University, Xiamen, China; ^4^Laboratory for Marine Fisheries Science and Food Production Processes, Laboratory for Marine Biology and Biotechnology, Pilot National Laboratory for Marine Science and Technology, Qingdao, China; ^5^School of Science and Medicine, Lake Superior State University, Sault Ste. Marie, MI, United States

**Keywords:** teleost fish, B cells, phagocytosis, cytokines, antigen presentation

## Abstract

The momentous discovery of phagocytic activity in teleost B cells has caused a dramatic paradigm shift from the belief that phagocytosis is performed mainly by professional phagocytes derived from common myeloid progenitor cells, such as macrophages/monocytes, neutrophils, and dendritic cells. Recent advances on phagocytic B cells and their microbicidal ability in teleost fish position B cells at the crossroads, bridging innate with adaptive immunity. Most importantly, an increasing body of experimental evidence demonstrates that, in both teleosts and mammals, phagocytic B cells can recognize, take up, and destroy particulate antigens and then present those processed antigens to CD4^+^ T cells to elicit adaptive immune responses and that the phagocytosis is mediated by pattern recognition receptors and involves multiple cytokines. Thus, current findings collectively indicate that teleost phagocytic B cells, as well as their counterpart mammalian B1-B cells, can be considered one kind of professional phagocyte. The aim of this review is to summarize recent advances regarding teleost phagocytic B cells, with a particular focus on the recognizing receptors and modulating mechanisms of phagocytic B cells and the process of antigen presentation for T-cell activation. We also attempt to provide new insights into the adaptive evolution of the teleost fish phagocytic B cell on the basis of its innate and adaptive roles.

## Introduction

It has become well-accepted that B cells in all vertebrates are functional antibody-secreting cells (ASCs) for the production of specific antibodies in response to certain invading foreign antigens and that they play vital roles in adaptive immunity (1). Phagocytosis is a specific form of endocytosis of phagocytes by which solid particles (including microbial pathogens) are internalized to form phagosomes and phagolysosomes, followed by antigen degradation to destroy the invaders or continued processing of antigenic information, eventually initiating adaptive immunity in vertebrates (2–4). Phagocytosis plays an essential role of linking the innate and adaptive immune responses in vertebrates. Classical phagocytosis is mainly accomplished by “professional” phagocytes, including macrophages/monocytes, neutrophils, and dendritic cells, but some “amateur” phagocytes (such as epithelial cells and fibroblasts) are able to engulf particulate antigens to a much lower degree in comparison to professional phagocytes (5). Although B cells are considered to be one of the three major professional antigen-presenting cells (APCs), it is well-recognized that they have the main responsibility of binding specific soluble antigenic peptides through B-cell receptors (BCRs) but do not phagocytose and present large non-specific particulate antigens. Therefore, the long-held paradigm is that B cells are non-phagocytic cells, even though evidence has been reported that CD5^+^ B-cell lymphoma was able to differentiate to macrophage-like cells (6). However, in 2006, Li et al. showed direct evidence for the first time in vertebrates that B cells derived from teleost fish and frog are capable of phagocytic and bactericidal activity through the formation of phagolysosome, a unique innate immunity that was previously only identified in professional phagocytes (7). Besides teleost fish, this novel phagocytic capability of B cells has also been extended into other vertebrates like reptiles (8), mice, and human (B1 subset) (9–13). Since then, numerous studies have been carried out in an attempt to elucidate the involvement of phagocytic B cells and their related novel aspects in both innate and adaptive immune responses, especially their evolutionary origins and the functional relationships between different B-cell subsets and macrophages. Details regarding those recent findings have been summarized and discussed in several excellent reviews (14, 15).

It is well-known that fish have both an innate and an adaptive immune system. Thus far, most of the elements of the innate immune system of higher vertebrates, as well as the counterpart molecules/receptors related to the mammalian adaptive immune system, including immunoglobulins, B-cell receptor (BCR), major histocompatibility complex class I and II (MHC I and MHC II), CD4, CD8, T cell receptor (TCR), etc., have also been identified in teleost fish (16). A variety of novel findings originally from studies on the fish immune system have led to major groundbreaking discoveries of previously unknown molecules and biochemical pathways involved in mammalian immunity (17–20). Due to the unique place of this fish on the evolutional timeline of life, the teleost fish has become an excellent non-classical animal model for exploring the evolutionary history of defense immune reactions in mammals (16, 21). As a vital facet of innate immunity, phagocytosis plays essential roles in bridging the innate and adaptive immune reactions in both teleost fish and mammalian species (22). The newly uncovered phagocytic and bactericidal capabilities of B cells not only lead to a paradigm shift for the fish immune system (7) but also open a new door for us to rethink the evolutionary structure and functional network as well as the underlying regulatory mechanisms of the current mammalian immune system. Increasing studies on phagocytic B cells indicated that the phagocytosis is mediated by a series of molecules related to innate and adaptive immunity (19). However, due to the limited availability of specific reagents for fish, the study on teleost phagocytic B cells is still at a very early stage, and more efforts are urgently required for further exploration of detailed immune functions in teleosts and in mammals as well.

In this review, we try to summarize the most recent advances in the following areas in relation to the phagocytosis of teleost B cells: (1) phagocytic B-cell subsets in teleost fish; (2) phagocytic receptors and related pathways involved in B-cell phagocytosis; (3) modulating cytokines in B-cell phagocytosis; (4) involvement of phagocytic B cells in antigen presentation; (5) effects of B-cell adaptive functioning (differentiation) on B-cell phagocytic capacity. We aim to better understand the innate roles of fish phagocytic B cells in interacting and activating their adaptive immune functions in the primitive vertebrate and hopefully to provide novel evolutionary insights for further elucidation of the interaction mechanisms of the innate and adaptive immune system in mammalian species.

## Phagocytic B-Cell Subsets in Teleost Fish

Until now, three different immunoglobulin isotypes (IgM, IgD, and IgT/Z), which are accordingly secreted by three major B-cell subsets (IgM^+^/IgD^+^, IgM^−^/IgD^+^, and IgM^−^/IgT^+^, or IgM^−^/IgZ^+^), have been identified and described in teleosts (23–26). IgM^+^ B cells (IgM^+^/IgD^+^ lineage), as well as the IgM, have been found as the major B cells and the most abundant immunoglobulin present in the serum of teleost fish, and these play crucial roles in fish systemic adaptive immunity (27–31). IgT/Z, secreted by the previously unknown IgT^+^ B cell (IgM^−^/IgZ^+^ lineage), which is a functional equivalent to IgA in mammals and birds, has also been demonstrated as a major player specialized in the teleost mucosa-associated lymphoid tissues (MALT), like intestine, skin, and gill, as well as in nasal-associated lymphoid tissue (NALT), pharyngeal mucosa, and buccal mucosa (32–36). The structural and functional specificity of IgT and IgT^+^ B cells, their important roles in mucosal immunity in teleost fish, as well as the potentially applicable aspects in aquaculture for developing novel strategies to prevent infectious diseases, have been reviewed elsewhere (26, 37). IgD has long been recognized as a co-expressed molecule maker with IgM on the surface of matured naïve IgM^+^/IgD^+^ B cells. More interestingly, a novel IgM^−^/IgD^+^ B-cell subset and two different types of secreted IgD have recently been characterized in two teleost species, catfish and trout, respectively; however, very little is known about the function of IgD and the two types of IgD^+^ B cells in teleost fish (38–40).

The phagocytic and bacteria-killing abilities of IgM^+^ B cells were originally discovered and characterized by Li et al. in rainbow trout (*Oncorhynchus mykiss*) and catfish (*Ictalurus punctatus*) (7). In their subsequent study, a previously unknown IgM^−^/IgT^+^ B-cell subset, which uniquely secretes IgT, was identified in rainbow trout, and it is also capable of phagocytic and microbicidal activity (41). With regard to IgD^+^ B cells, the involvement of surface IgD in the phagocytic activity of IgM^+^/IgD^+^ B cells is still unclear, and thus far, no reports are available that address the phagocytic ability of the newly identified IgM^−^/IgD^+^ B cells in catfish and trout. However, the evidence that IgD^+^/IgM^−^ plasmablasts constitute a major lymphocyte population in the teleost intestine and gills implies that the IgD^+^ cells may play potential roles in MALT (42).

The unavailability of specific monoclonal antibodies (mAbs) or B-cell markers is a major barrier that has slowed down the exploration of the phagocytic activities of teleost fish B cells (16). In recent years, the number of reports on the phagocytic B cells of teleost fish has been rapidly increasing. In addition to rainbow trout and catfish, as shown in Table 1, phagocytic B cells from about 10 different teleost fish species have been identified. It needs to be pointed out that most of these studies were only focused on IgM^+^ B-cell subsets due to the shortage of specific mAbs against IgT or IgD in these fish species. In these investigations, different functions of the teleost fish B cells were revealed in adaptive immunity or innate immunity. Although the IgM^+^ B cells are capable of phagocytosis, their phagocytic capabilities differ significantly in different fish species (15, 43). For example, Overland et al. demonstrated very varied phagocytic activities by incubating fluorescent beads with IgM^+^ B cells derived from either head kidney (HKL) or peripheral blood (PBL) of Atlantic salmon (*Salmo salar* L.) and cod (*Gadus morhua* L.), respectively (43). Similarly, highly variable phagocytic abilities for the IgM^+^ B cells to ingest microbeads or different microbial particles were also observed in zebrafish (*Danio rerio*), lumpfish (*Cyclopterus lumpus* L.), half-smooth tongue sole (*Cynoglossus semilaevis*), large yellow croaker (*Larimichthys crocea*), turbot (*Scophthalmus maximus*), and Japanese flounder (*Paralichthys olivaceus*) (46, 52, 61). It is worth noting that various factors, as well as those mentioned above such as fish species and different immune organs/tissues, should also be seriously considered during phagocytic activity assay, for example, the physiological status of the fish, the size and nature of target particles, and the methods applied to incubate phagocytic B cells with various particles (mainly including the ratio of B cells to target particles, the opportunity for targets to interact with B cells, and the duration of incubation) (7, 53). In addition, the phagocytic process in both mammals and teleost B cells can be inhibited in a dose-dependent manner by cytochalasin B and colchicine, which indicates the involvement of cellular microtubules and microfilaments in B cells to internalize particles and bacteria (7, 10, 12, 41).

**Table 1:** Studies of phagocytic B cells in teleost fish from 2006 until now.

**Time**	**Species**	**B-cell subsets**	**Phagocytic ability**	**Microbicidal ability**	**Antigen-presenting ability**	**References**
2006	*Oncorhynchus mykiss* *Ictalurus punctatus*	IgM^+^ IgM^+^	YES YES	YES YES	NA NA	(7)
2010	*Oncorhynchus mykiss*	IgM^+^ and IgT^+^	YES	YES	NA	(41)
	*Salmo salar* L.	IgM^+^	YES	NA	NA	(43)
	*Gadus morhua* L.	IgM^+^	YES	NA	NA	(43)
2013	*Danio rerio*	IgM^+^	Little	Little	NA	(44)
2014	*Danio rerio*	IgM^+^	YES	YES	YES	(45)
2015	*Cyclopterus lumpus* L.	IgM^+^	YES	NA	NA	(46)
2016	*Oncorhynchus mykiss*	IgM^+^	YES	NA	YES	(47)
2017	*Oncorhynchus mykiss*	IgM^+^	YES	YES	NA	(48)
	*Oncorhynchus mykiss*	IgM^+^	YES	NA	YES	(49)
	*Oncorhynchus mykiss*	IgM^+^ and IgT^+^	YES	YES	NA	(50)
	*Oncorhynchus mykiss*	IgM^+^	YES	NA	NA	(51)
	*Cynoglossus semilaevis*	IgM^+^	YES	NA	NA	(52)
2018	*Scophthalmus maximus*	IgM^+^	YSE	NA	NA	(53)
	*Paralichthys olivaceus*	IgM^+^	YES	YES	NA	(54)
	*Oncorhynchus mykiss*	IgM^+^	NA	NA	YES	(55)
2019	*Oreochromis niloticus*	IgM^lo^ and IgM^hi^	YES	YES	NA	(56)
	*Oncorhynchus mykiss*	IgM^+^	YES	YES	YES	(57)
	*Oncorhynchus mykiss*	IgM^+^	YES	YES	YES	(58)
	*Paralichthys olivaceus*	IgM^+^	YES	YES	NA	(59)
	*Paralichthys olivaceus*	IgM^+^	YES	YES	YES	(60)
	*Larimichthys crocea*	IgM^+^	YES	NA	NA	(61)

*“NA” indicates no data; “YES” indicates that the cells have the function; “Little” indicates there is little phagocytosis of B cells*.

## Phagocytic Receptors to Initiate B-Cell Phagocytosis

**Figure 1:**
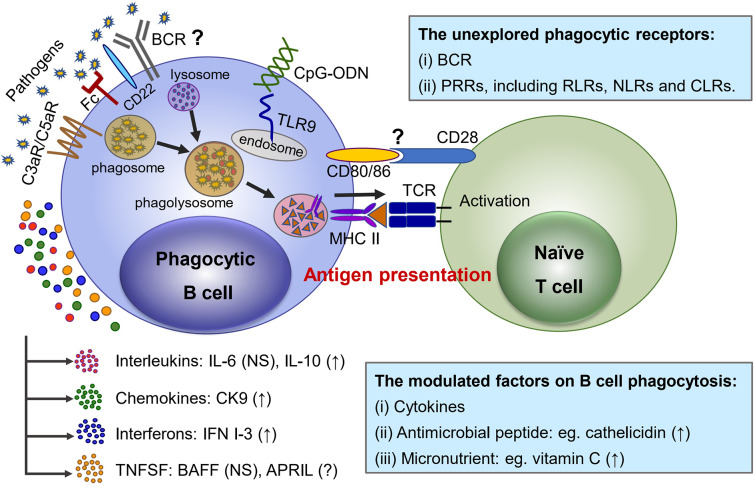
Phagocytic receptors and modulating factors functioning in the phagocytosis as well as the antigen-presenting effect to naïve T cells in teleost phagocytic B cells. (1) Fc, C3a, and C5a receptors presenting at the surface of teleost B cells are involved in phagocytosis. CD22 plays regulatory roles in the micropinocytosis-dependent pathway to internalize large particles by turbot and Japanese flounder IgM^+^ B cells, but the contribution of BCR remains to be further studied. The PRRs recognize a wide variety of PAMPs to initiate phagocytosis, and TLR9 ligation is mediated by CpG ODN, resulting in the phagocytic capacities of splenic IgM^+^ B cells being up-regulated. Other PRRs, including RLRs, NLRs, and CLRs, remain to be explored. (2) Some cytokines are involved in the phagocytosis regulation of teleost B cells; specifically, IL-10, CK9, and IFN I-3 up-regulate phagocytosis, while IL-6 and BAFF have no significant effect. Other factors, such as antimicrobial peptide (cathelicidin) or micronutrients (vitamin C), have been proved to have an up-regulation function on teleost B-cell phagocytosis. (3) Pathogens (such as bacteria and viruses) are recognized by teleost B cells and engulfed into a vesicle as phagosome, with subsequent formation of maturated phagolysosome with a lysosome. The phagocytic B cells digest the internalized contents into component parts and proceed to antigen presentation with MHC II, which activates naïve T cells. However, no direct evidence has yet clarified how CD80/86 interact with CD28 in naïve T cells when B cells proceed to antigen presentation. “?” means as yet unknown; “NS” indicates no significant effect; “↑” means up-regulation.

Similar to professional phagocytes, it has been clearly evidenced that both IgM^+^ and IgT^+^ B cells of rainbow trout phagocytose and kill bacteria through engulfment of target particles into phagosome and subsequent formation of maturated phagolysosome (Figure 1), and a similar actin polarization internalizing process has also been demonstrated in phagocytic B cells (7, 45). However, the involvement of functional receptors on the surface of phagocytic B cells for initial recognition of and interaction with certain molecules of target particles, as well as the difference from professional phagocytes, is not yet well-understood. Li and his colleagues demonstrated that the phagocytic activity of IgM^+^ and IgT^+^ B cells could be significantly enhanced once the target bacteria had been opsonized with antiserum or complement, which indicated a similar involvement of Fc receptor and complement receptors in the phagocytosis of both B cells and professional phagocytes (7, 45). Moreover, solid evidence has also confirmed the presence of C3a and C5a receptors on the surface of trout IgM^+^ B cells and also on granulocytes (62–64). In addition, significant enhancement of C3d-linked target particles being phagocytosed by trout IgM^+^ B cells indicated the presence of a mammalian CR2-like receptor (C3d receptor) on the surface of fish B cell (65). Similar phenomena that up-regulated phagocytosis were discovered in mouse IgM^+^ B cells after incubation with complement-opsonized target particles (11). The cooperation of complement and phagocytic B cells both in teleost and mammalian species indicates the essential importance of B cells in the linkage of innate and adaptive immunity (Figure 1).

Their obvious difference from macrophages is that teleost B cells express B-cell-specific markers, including mIgM, CD79a, and CD79b, which constitute the BCR complex (66). BCR is crucial not only for specific binding of foreign antigens but also for signal transduction and the downstream regulation of B-cell activation and differentiation. Primary human B cells have shown the ability to uptake live *Salmonella* but not dead ones through BCR (67), but it remains to be clarified whether the internalizing process is a BCR-mediated or bacteria-mediated mechanism on this occasion. It has been demonstrated that phagocytosis of murine B1-a and B1-b B cells derived from the peritoneal cavity is BCR-independent (12). However, there was a report that *bcr*-transgenic mice whose B cells expressed more BCR exhibited 3-fold higher phagocytic activity than littermate control mice, which suggested that the transgenic BCR might promote B-cell phagocytosis (10). Regarding teleost B cells, we recently identified a co-stimulatory signal molecule that is equivalent to mammalian B cell-associated receptor (CD22) in Japanese flounder (54). The CD22-like molecule can not only provide a co-stimulatory signal for activation of IgM^+^ B cells but also play essential regulatory roles in the macropinocytosis-dependent pathway principally relied upon by turbot and Japanese flounder IgM^+^ B cells to internalize large particles (53, 54). This finding implies that teleost BCR, associated with its co-receptors, might be a crucial mediator in relation to B-cell phagocytosis as shown in mammals. Although the macropinocytosis-dependent pathway of turbot and Japanese flounder IgM^+^ B cells likely implies the existence of another non-receptor-mediated endocytosis pathway in teleost IgM^+^ B cells (53, 54), the regulation of CD22 in macropinocytosis-dependent endocytosis seems to indicate that macropinocytosis is regulated by other receptors instead of BCR. Thus, further studies are necessarily required to figure out the contribution of BCR as well as other co-receptors to B cells in ingesting large particulate antigens.

Due to being responsible for pattern recognition receptors (PRRs) that recognize a wide variety of pathogen-associated molecular patterns (PAMPs) to initiate phagocytosis (68), besides the abovementioned receptors, other cell surface molecules (receptors) especially the common PRRs identified on professional phagocytes, such as Toll-like receptors (TLRs), Retinoic acid-inducible gene (RIG)-I-like receptors (RLRs), NOD-like receptors (NLRs), and C-type lectin receptors (CLRs), may also be involved in B-cell phagocytosis (69). Thus, far, few studies of PRRs are available in relation to the phagocytosis of teleost B cells.

TLRs, a family of single, non-catalytic, membrane-spanning PRRs, are responsible for pathogen sensing by recognizing specific PAMPs and then activating signaling cascades to trigger innate immunity (70). In rainbow trout, multiple TLR genes were analyzed in IgM^+^ B cells, which suggested an important role for B cells in triggering the innate immune function (71). Particularly, a CpG oligodeoxynucleotides (ODN)-mediated TLR9 ligation has been described for the up-regulation effect on phagocytic capacities of splenic IgM^+^ B cells in Atlantic salmon and rainbow trout (57, 72). In addition, RLRs, NLRs, and CLRs have been identified in teleost fish with similar antiviral or antibacterial immune functions as in mammals (73–78); however, there is not yet any direct evidence to show their regulatory effects on teleost B-cell phagocytosis (Figure 1).

## Fish Cytokines Modulating B-Cell Phagocytosis

Cytokines are produced by various immune cells, including professional phagocytes, and can be classified as interleukins, chemokines, interferons, tumor necrosis factors (TNFs), and growth factors on the basis of their structure and function (79). To date, all of the major cytokine families existing in mammals have been found in teleost fish, and they play important roles in regulating hematopoiesis, inflammation, and adaptive immunity (80). Numerous investigations have been carried out in an attempt to elucidate the regulation mechanisms of fish cytokines in both innate and adaptive immune responses; here, we only review the regulating roles of certain cytokines in the phagocytosis of teleost B cells (Figure 1).

Interleukins are intercellular cytokines, and, to date, the regulatory mechanisms of interleukin-6 and−10 (IL-6 and IL-10) in the phagocytic activity of teleost IgM^+^ B cells have been recognized (47, 59). Studies have indicated that IL-6 has no effect on the phagocytic activity of rainbow trout IgM^+^ B cells (47), whereas IL-10 could enhance the phagocytosis of IgM^+^ B cells in flounder (59). Moreover, IL-10R and STAT3 have been found to be involved in the regulation of IL-10-stimulated phagocytosis (59). The positive effect of IL-2 on phagocytosis has been explored in myeloid-origin cells in rainbow trout; however, a divergent effect was observed on PBL lymphoid cells, even though its effect on B lymphocytes was not separately investigated (81). Other interleukins have been cloned and characterized in teleost fish, but the effects on teleost B-cell phagocytosis have not yet been explored (18).

Chemokines consist of a superfamily of small proteins (8–10 kDa) that are involved in a variety of immune and inflammatory responses. In general, they act primarily as chemoattractants and activators for recruiting specific types of leukocytes (82). CK9, a CC chemokine in rainbow trout (resembling mammalian CCL25), has been shown to have a strong chemotactic capacity and to up-regulate phagocytic capacity for both IgM^+^ B cells and macrophages (83). Though many chemokines have been identified in rainbow trout, functional study of B-cell phagocytosis is still limited. For example, for CK11, its antimicrobial activity rather than its phagocytic activity has been recovered (84).

Interferons (IFNs) are a group of proteins that are made and released by host cells in response to intracellular pathogens (such as viruses, bacteria, or parasites) (85). A recent study indicated that type I interferon-3 (IFN I-3) significantly enhanced phagocytosis of IgM^+^ B cells for *Lactococcus lactis* and *Edwardsiella tarda* (60). However, no other interferons have been explored for their roles in the phagocytosis of teleost B cells.

The TNF ligand superfamily (TNFSF) represents a multifunctional proinflammatory cytokine that activates signaling pathways for cell survival, apoptosis, inflammatory responses, and cellular differentiation (86). More recently, B cell-activating factor (BAFF), a proliferation-inducing ligand (APRIL), and BAFF-APRIL-like molecule (BALM), as well as the BAFF receptor (BAFF-R) and other related molecules, were identified in rainbow trout (49, 55, 87, 88). However, a recent study indicated that BAFF did not alter the phagocytic activity of IgM^+^ B cells (49). In regard to APRIL or BALM, their function in B-cell phagocytosis in teleosts remains to be further investigated.

Interestingly, cathelicidin, a kind of antimicrobial peptide, was found to be able to significantly facilitate the phagocytic, intracellular bactericidal, and reactive oxygen species activities in trout IgM^+^ and IgT^+^ B cells (50), a phenomenon that has been well-characterized previously in macrophages. These findings provide new evidence in support of the close relationship between B cells and macrophages in vertebrates. Additionally, vitamin C, an essential micronutrient, has also been reported to significantly increase the phagocytosis activity of teleost IgM^+^ B cells from head kidney when pre-incubated, while co-incubation has no obvious effect (51). Although Vitamin C does not affect cytokine expression (including IL-1β, IL-8, COX-2B, TNF-α, cathelicidin 2, and hepcidin) of head kidney leukocytes, the impact on IgM^+^ B cells remains unknown. Whether vitamin C acts via modulating the transcriptome of cytokines to regulate IgM^+^ B-cell phagocytic activity, like cathelicidin, which improves the phagocytosis of IgM^+^ B cells (50), needs to be explored further.

## Involvement of Phagocytic B Cells in Antigen Presentation

Phagocytosis not only provides a critical first line of defense against invading pathogens but is also a very efficient mechanism for antigen presentation in order to link innate with adaptive immune processes. Professional phagocytes (macrophages and dendritic cells) and B cells have long been recognized in higher vertebrates as professional APCs that provide antigenic ligands to activate T cells (22). Among them, professional phagocytes are generally characterized as having high efficiency in ingesting and destroying internalized pathogens, followed by effective presentation of antigens to both CD4^+^ and CD8^+^ T cells (2, 4), whereas B cells mainly process soluble antigens and are restricted to loading antigens onto MHC II and eventually presenting antigens to CD4^+^ T cells (89). Currently, phagocytosis and bactericidal abilities have been identified in teleost B cells as well as in mammalian B1-B cells (7, 10–12), and the next to be expected is that a previously unrecognized function of presenting internalized particulate antigens to elicit T cells will be revealed. It was first demonstrated in mammals that the phagocytic B1-B cells derived from the murine peritoneal cavity, liver, or spleen have the ability to present antigen to CD4^+^ T cells, which indicated that B1-B cells are a kind of APC and are able not only to present soluble antigens but also to effectively present ingested large particulate antigens (Figure 1) (10–12, 67). Similarly, the phagocytic IgM^+^ B cells in zebrafish have also been proved to act as pivotal initiating APCs (similar to dendritic cells) that prime adaptive immunity when presenting both soluble antigens and bacterial particles to prime naïve CD4^+^ T cell proliferation, which was mediated by MHC II and costimulatory molecules (CD80/CD86 and CD83) (45). In addition, indirect evidence of significant up-regulation of antigen-presenting-capacity related genes, including MHC II, as well as CD83 and CD80/86, in phagocytic IgM^+^ B cells have been described in a number of teleost species in response to various pathogenic bacteria or viruses, which indicates that teleost IgM^+^ cells act as APCs during the course of a pathogenic infection (56, 90, 91). However, the ability of phagocytic B cells to cross-present particulate antigens to CD8^+^ T cells is unknown and needs to be investigated in the future in both mammals and teleost fish.

In addition, it needs to be pointed out that the strikingly high number of B cells in teleost fish blood combined with their significantly high phagocytic and intracellular killing abilities implies that fish phagocytic B cells may play more important roles in effective antigen presentation than mammalian B cells (7, 12).

## B-Cell Differentiation and Phagocytic Capacity

Apart from the different B-cell subsets, which variously express IgM, IgD, or IgT/Z, in teleost fish, attention has also been paid to the phagocytic activity of teleost B cells at different developmental and differentiation stages (44, 92). Unlike for mammals, there are no specific antibodies available to distinguish teleost B cells precisely on their developmental/differentiation status, which hinders further exploration of the effects on their phagocytic function. However, examining the expression levels of B-cell-specific transcription factors can provide a comparative approach for studying teleost B-cell development (93). Thus, far, Paired box-5 (Pax5), Early B cell Factor-1 (EBF1), B lymphocyte-induced protein-1 (Blimp1), and X-box protein I (XbpI) have been identified in rainbow trout and applied to differentiate resting B cells, activated B cells, plasmablasts, and plasma cells (94–96). Pax5 is expressed from pre-B cells to mature B cells, while Blimp1 is a master regulator of terminal B cells by down-regulating the expression of Pax5 and leading to the maturation of plasmablasts to plasma cells (92). Significantly higher expressions of Pax5 and Blimp1 have been demonstrated in resting IgM^+^ B cells than in IgM^−^ cells (71, 97, 98). However, Pax5 is usually suppressed by its repressor, Blimp1, in activating B cells, leading to shifts in the expression of immunoglobulin from membrane to secreted forms (99). In rainbow trout, the IgM^+^ B cells from the peritoneal cavity have been proposed to be classifiable as IgM^hi^ or IgM^lo^ B cells on the basis of membrane IgM (mIgM) concentration, respectively (92). Moreover, they can also be distinguished as either a naïve B-cell subset or an antibody-secreting subset depending on the expression levels of Pax5 and Blimp1 genes (58). Similarly, in our recent study, IgM^hi^ and IgM^lo^ B cells, which resemble naïve/mature B cells and plasma-like cells, respectively, have also been described in the peripheral blood of Nile tilapia (*Oreochromis niloticus*) (56, 100). We demonstrated that B-cell differentiation may cause a decrease in phagocytic capacity but not in phagocytic ability of phagocytic IgM^+^ B cells in Nile tilapia (56). Moreover, MHC II expression was significantly higher in the phagocytic IgM^hi^ B cells than in the IgM^lo^ B cells, implying that a variable antigen-presentation capacity exists in IgM^+^ B cells under different differentiation status (56). The change in the phagocytic activity in teleost B cells following the differentiation process seems in line with the shift in their specialized function of secreting antibody in humoral immunity. It is well known that mammalian B2-B cells comprise the major portion of peripheral blood B cells and are specialized as antibody-secreting B cells; whether similar functional B-cell subsets exist in teleost fish remains to be investigated.

## Concluding Remarks and Prospective Directions

Phagocytosis is the first line of defense of the immune system to eliminate most invading pathogenic microorganisms and is an essential part of tissue homeostasis and remodeling, which offers protective defense to bodies. Phagocytic B cells are capable of taking up distinct microbial pathogens by interacting with different receptors through related regulatory pathways, subsequently presenting the antigen to prime naïve T cells, and finally initiating B cell differentiation to secret specific antibodies. Comparative studies on such phagocytic activity of B cells from different species have also dug deeper to understand their origin and the evolutionary and functional relationships of mammalian B cells and macrophages. However, current studies also raise concerns that may be addressed by future prospective studies on teleost phagocytic B cells, such as: (1) whether there are any different receptors required by phagocytic B cells as opposed to professional phagocytes (macrophages, neutrophils, and dendritic cells) in responding to different microorganisms (species, and/or virulence); (2) whether any different regulating mechanisms and pathways are involved in the phagocytic and antigen-presenting process in B cells as opposed to the professional phagocytes; (3) how the phagocytic B cells modulate and deal with differently sized, specific and non-specific, particulate and soluble antigens with/without BCR to generate the specificity of the finally produced antibodies? Further investigations to address the above concerns will not only provide new insights into the immune defense regulations of phagocytic B cells in teleost fish but will also enable a better understanding of the evolution and origin of the mammalian immune system. Particularly, it is of great interest to explore teleost phagocytic B cells and their regulatory mechanisms so as to facilitate the development of novel and more effective strategies to prevent infectious diseases in the aquaculture industry.

## Author Contributions

JL, LW, and ZQ wrote the manuscript. LL, HL, and JY contributed with suggestions, discussions, and critical reading of the manuscript. JY and JL designed the contents of this paper.

## Conflict of Interest

The authors declare that the research was conducted in the absence of any commercial or financial relationships that could be construed as a potential conflict of interest.
